# Electron work function–a promising guiding parameter for material design

**DOI:** 10.1038/srep24366

**Published:** 2016-04-14

**Authors:** Hao Lu, Ziran Liu, Xianguo Yan, Dongyang Li, Leo Parent, Harry Tian

**Affiliations:** 1 Dept. of Chemical and Materials Engineering, University of Alberta, Edmonton, Alberta, T6G 1H9, Canada; 2 School of Mechanical Engineering, Taiyuan University of Science and Technology, Taiyuan, 030024, People’s Republic of China; 3 Suncor Energy, Fort McMurray, Alberta, T9H 3E3, Canada; 4 Metallurgical/Materials R&D, GIW Industries, Grovetown, GA 30813-2842, USA

## Abstract

Using nickel added X70 steel as a sample material, we demonstrate that electron work function (EWF), which largely reflects the electron behavior of materials, could be used as a guide parameter for material modification or design. Adding Ni having a higher electron work function to X70 steel brings more “free” electrons to the steel, leading to increased overall work function, accompanied with enhanced *e*^−^–*nuclei* interactions or higher atomic bond strength. Young’s modulus and hardness increase correspondingly. However, the free electron density and work function decrease as the Ni content is continuously increased, accompanied with the formation of a second phase, *FeNi*_3_, which is softer with a lower work function. The decrease in the overall work function corresponds to deterioration of the mechanical strength of the steel. It is expected that EWF, a simple but fundamental parameter, may lead to new methodologies or supplementary approaches for metallic materials design or tailoring on a feasible electronic base.

With rapid advance in material technology, material design has been required to rely on more fundamental principles. The intrinsic mechanical behavior of metallic materials is largely governed by their electron behavior, which determines the atomic bond strength^1^. Great effort has long been made to correlate mechanical properties of materials to their electron configurations based on quantum mechanics^2^. However, quantum theories are generally complicated and difficult to be used in material design, especially for structural materials which often contain various phases. Thus, it is highly desired to have simple but fundamental parameters which largely reflect the electron behavior of metallic materials and could be used in material design in a feasible manner.

Considerable research has demonstrated that electron work function (EWF) could be such a parameter for the above-mentioned purpose. EWF is the minimum energy required to move electrons at Fermi level inside a metal to its surface without kinetic energy^3^. Work function of a material is determined by its composition and the charge redistribution on its surface caused by a dipole layer^4^,^5^. It is one of the fundamental electronic properties of metals, depending on both the bulk composition and surface condition. EWF largely reflects the electron behavior of materials and is also influenced by the surface condition, such as adsorption^6^, contamination^7^, surface roughness and corrosion products^8^, etc. Although the work function is influenced by the surface condition, it fundamentally reflects the atomic interaction and is directly related to bulk properties^9^,^10^,^11^. This parameter could thus be used to predict and evaluate mechanical properties of metals. Furthermore, EWF is valuable not only for in-depth understanding of materials but also for developing alternative or supplementary approaches for material design.

Previous studies have demonstrated that EWF is intrinsically related to many mechanical properties of pure metals, such as Young’s modulus, hardness, yield strength and thermal expansion, etc^12^,^13^,^14^. These relationships can be extended to isomorphous solid solutions^15^. It has been demonstrated that EWF provides a new and effective approach for alloying element selection to achieve adjustable strength for isomorphous solid solutions. However, realistic industrial materials generally have multi-phase microstructures. Thus, whether or not the established relationships between EWF and mechanical properties of pure metals are applicable to multi-phase alloys needs to be investigated. The current research efforts are oriented to determination of the relation between EWF and mechanical properties of multi-phase alloys and explore application of EWF for realistic material design. In this study, X70 steel (0.066%C) is used as a sample base alloy, to which Ni (*ϕ*_Ni_ = 5.15 *eV* vs. *ϕ*_*Fe*_ = 4.5 *eV*^16^) is added in order to study how the work function and corresponding mechanical properties change with respect to %Ni and the formation of second phases. The possibility of using EWF for multiphase alloy modification is discussed.

## Results and Discussion

### Changes in EWF and mechanical properties with %Ni

**Figure 1:**
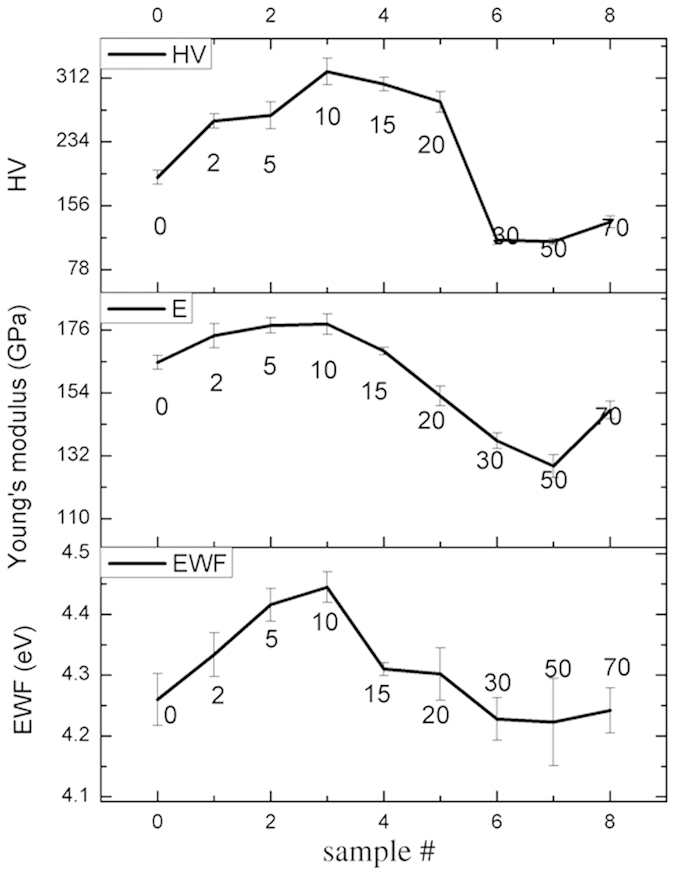
EWF, Young’s modulus and hardness of X70 samples with different concentrations of Ni. The numbers around each curve represent %Ni (e.g., 15 stands for 15%Ni), x-axis gives I.D. number for the samples (e.g., sample 2 has its %Ni = 5%). EWF was measured using a Scanning Kelvin Probe (SKP); Young’s modulus and hardness were determined using a micro-indenter.

Measured EWF, Young’s modulus and hardness of samples with different concentrations of Ni are given in Fig. 1. As shown, values of these properties increase initially with %Ni and reach maxima at 10%Ni, however, when more than 10% Ni is added, values of all the properties decrease. The changes in Young’s modulus and hardness well follow the changes in EWF. In order to ensure that the measured reflected average mechanical properties of bulk materials, macro-hardness of the samples was also evaluated using a macro-hardness tester. Result of the measurement is given in Table 1. As shown, the trend of the macro-hardness with respect to %Ni is consistent with that of results obtained from micro-indentation testing.

The initial increase in Young’s modulus with EWF as a small amount of Ni was added to X70 can be explained based on the fact that the electron work function reflects the stability of the electron state and the resistance to breaking of atomic bonds. A metal which has a higher electron work function requires more energy to change its electron state. Adding an element with higher work function, the modified base alloy would have an increased work function. This consequently generates higher barriers to attempts to change its mechanical and electrochemical states, which are related to the electron state that governs the atomic bond strength. When Ni is added to X70 steel, the overall EWF increases, corresponding to a stronger bonding between adjacent Ni and Fe atoms or a larger barrier to separation of a Fe-Ni atomic pair, as a result, Young’s modulus of the steel increases. As for hardness, the stronger atomic bonding also raises barriers to generation and movement of dislocations, leading to higher hardness. It should be indicated that the increase in hardness is less intrinsic, since it is also influenced by other factors such as solid-solute strengthening through the process of pinning dislocations by added solid solute^17^. However, although hardness is a property influenced by multiple factors such as elastic modulus, slip systems, and the activation energy of dislocations, etc., the atomic bonding is a primary intrinsic factor. Or in other words, for metals having the same crystal structure, their hardness is mainly governed by the atomic bond strength and thus the elastic modulus, which is therefore correlated to EWF^13^. The activation energies for dislocation generation and movement motion are also related to the elastic modulus and thus the electronic state or EWF. More details about the relation between EWF and hardness can be found in ref. 13, which shows the following correlation between hardness and EWF: *H* (1 − *υ*^2^) ∝ *φ*^6^, where *υ* and *φ* are poisson’s ratio and work function, respectively. This explains why the variation in hardness with %Ni shows a trend similar to those of Young’s modulus and EWF as illustrated in Fig. 1. Clearly, the hardness is correlated to the work function.

**Figure 2:**
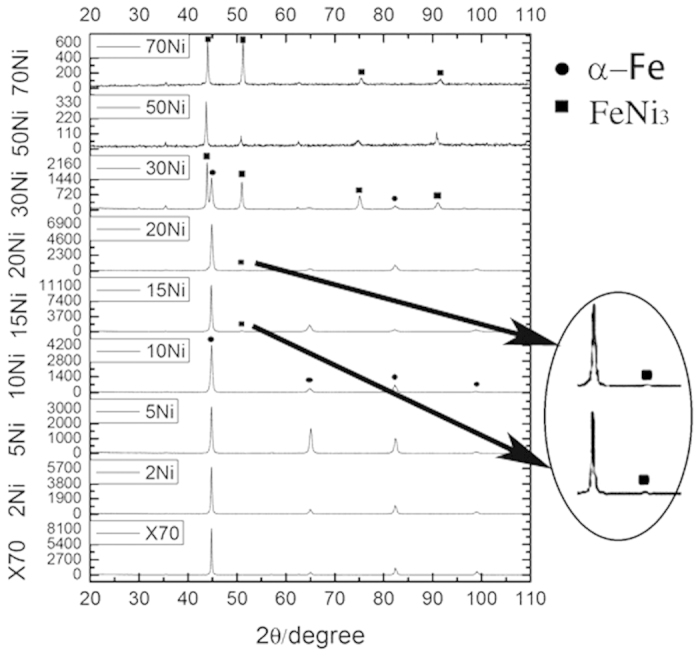
XRD patterns of X70-Ni samples containing different amounts of Ni addition. When Ni concentration is lower than 10%, samples show only one phase. Once the amount of Ni is more than 10%, samples contain two different phases, ferrite and *FeNi*_3_. However, X70-70Ni basically only shows peaks of *FeNi*_3_.

However, when more than 10% nickel was added to X70 steel, both EWF and the mechanical strengths decreased. Such changes are different from the effect of a small amount of Ni addition on the steel and also what previous studies showed, i.e. adding an element with a higher work function can raise the overall work function and the mechanical strength of isomorphous alloys^15^. In order to clarify the discrepancy, possible changes in structure or microstructure were examined through X-ray diffraction (XRD) analysis. Obtained XRD patterns of X70 samples with different amounts of added Ni are illustrated in Fig. 2. As shown, below 10%Ni, samples are homogeneous without second phases present. When 15 wt% Ni is added to X70, very small peaks of FeNi_3_ phase show up. The peaks of FeNi_3_ become much stronger when 30 wt% Ni is added to X70. When 70 wt% Ni is added, the sample contains almost FeNi_3_fully, which is consistent with the Fe-Ni phase diagram and observations by others^18^,^19^. Thus, when the second phase forms in the samples, one cannot simply expect that the mechanical strength of the alloy is enhanced by alloying with an element that has a higher work function.

It should be pointed out that FeNi, Fe_3_Ni and *γ* - Fe all have FCC structures and their XRD peaks are close to or overlap with those of FeNi_3_. However, we believe that the formed phase is FeNi_3_ rather than either FeNi or Fe_3_Ni based on the phase stability and mechanical properties. According to extensive studies^18^,^20^, the diffusion rate of Ni in Fe-Ni system is very low. Thus, some phases shown in the Fe-Ni phase diagram are hardly to obtain or be observed experimentally. FeNi_3_ is a stable intermetallic phase in the Fe-Ni system, while FeNi and Fe_3_Ni are metastable phases according to both experimental and theoretical (enthalpy and free energy) studies^21^,^22^,^23^. Thus, FeNi_3_ is a favorite phase, compared to the metastable FeNi and Fe_3_Ni. The metastable phases may exist in very fine particles of Fe-Ni alloys when the cooling rate is high^24^. FeNi and Fe_3_Ni are indeed observed in Fe-Ni nanopaticles but not in bulk materials fabricated in laboratory. Besides, bulk modulus of FeNi_3_ is lower than that of Fe and the latter is lower than those of FeNi and Fe_3_Ni^25^. We performed nano-indentation tests to evaluate hardness of the formed second phase domains in the X70-30Ni sample and confirmed that the formed phase was softer than X70 which is basically *α*-Fe. Based on the above analysis, we believe that the phase marked with black squares in XRD patterns is FeNi_3_ rather than metastable FeNi or Fe_3_Ni.

**Figure 3:**
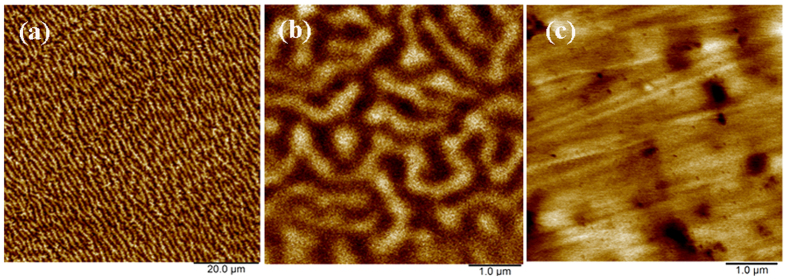
(**a**) a magnetic map of X70-50Ni sample with strip pattern over an entire area of 80 × 80 *μm*, (**b**) a close view of the magnetic map over a smaller area, and (**c**) a corresponding work function map of the area shown in (**b**), in which the dark domains have lower work function and are believed to be FeNi_3_ precipitated in *α*-Fe matrix (the bright area of the EWF map).

The phase marked with black squares in the XRD patterns should not be *γ*-Fe either, since the formed phase is softer than *α*-Fe, while *γ*-Fe is harder with higher EWF than *α*-Fe. Thus, the formed softer phase cannot be *γ*-Fe. In order to further confirm this conclusion, we conducted magnetic mapping for X70-50Ni sample with magnetic force microscopy (MFM) using a multimode atomic force microscope. Similar to *α*-Fe, FeNi_3_ is a ferromagnetic phase, while γ-Fe is a paramagnetic phase^21^,^23^. In the magnetic map, a ferromagnetic phase shows a strip pattern while the paramagnetic γ-Fe phase does not have such pattern^26^. We have examined samples under study, no paramagnetic γ-Fe domains were observed and all obtained magnetic maps only show typical ferromagnetic features in the entire sample area under analysis. Figure 3(a) shows a representative magnetic map of a X70-50Ni sample with a strip pattern over an area of 80 × 80 *μm*. No paramagnetic γ-Fe domains are presented. Figure 3(b,c) present a closer view of the magnetic map and a corresponding work function map, which shows the presence of sub-micron ferromagnetic domains that are softer with lower EWF than the matrix. These suggest that no detectable γ-Fe existed in the sample under examination.

**Figure 4:**
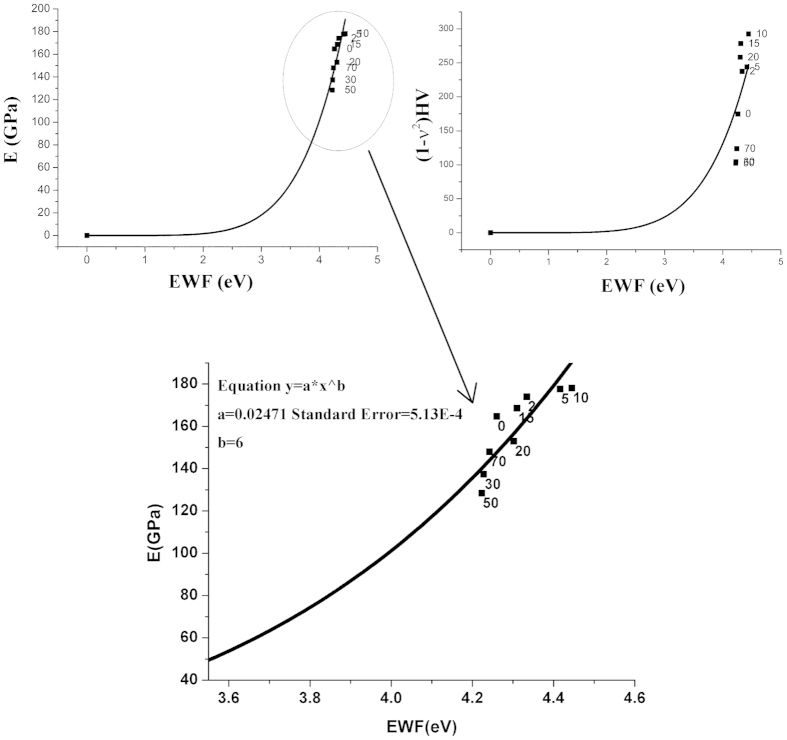
Variations in Young’s modulus (E) and hardness (H) with EWF. Curves of E ~ EWF and (1 − *υ*^2^) H ~ EWF of Ni-added X70 samples were obtained by fitting experimental data points. *υ* is the passion’s ratio. The curves are consistent with theoretical sixth power relationships with similar coefficients (*β*).

Previous studies have demonstrated that EWF has sixth power relationships with Young’s modulus and hardness of metals^12^, e.g., *E* = *βφ*^6^, where E is the Young’s modulus or elastic modulus and *β* is a coefficient. Such relationships still exist in the present case (See Fig. 4), no matter whether the second phase, *FeNi*_3_, exists or not. A fitted coefficient (*β*) for *E*~*φ* curve shown in Fig. 4 equals to 2.47 × 10^−2^, which is consistent with previous theoretical analysis^12^.

**Figure 5:**
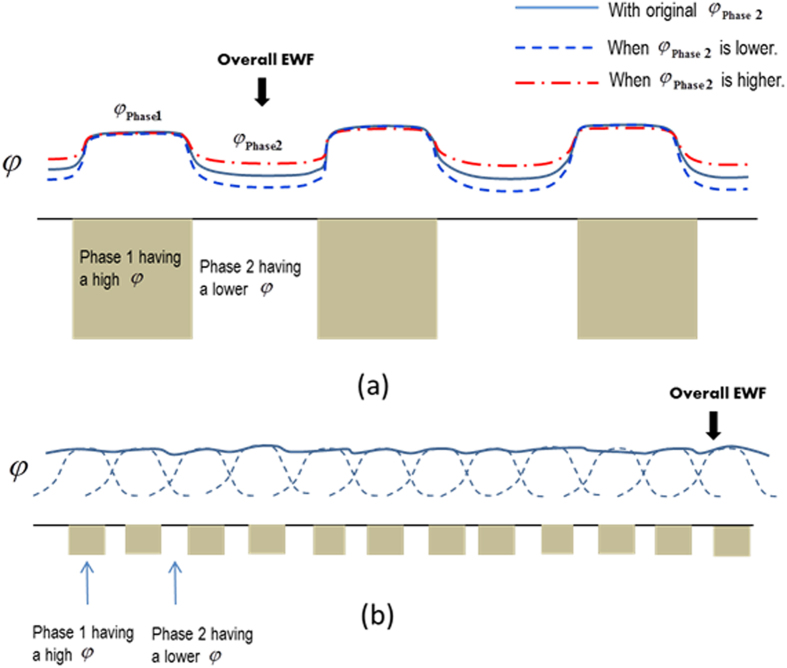
Schematic illustrations: effects of EWFs of individual phases and microstructure on overall EWF of a two-phase material. (**a**) The overall EWF is mainly the EWF of the phase having a lower EWF; (**b**) The overall EWF increases as the size of the phase with a higher EWF becomes smaller and more densely distributed.

It appears that the overall mechanical properties are determined by the overall work function to a certain degree. Figure 5 schematically illustrates two general cases to show how the individual phases with different scales affect the overall EWF, resulting in possible influences on the overall mechanical properties of the material systems. Figure 5(a) shows a two-phase material in which phase 1 has a higher EWF than phase 2 (softer). When EWF of the material is measured under the applied potential, electrons are pulled out basically from phase 2 with a lower EWF (*φ*_Phase2_). Thus, the overall EWF is dominated by *φ*_Phase2_. The overall mechanical strength is lowered by the softer phase 2, corresponding to a lower overall EWF. The situation is reversed if phase 2 becomes harder with a raised EWF. Thus, variations in the overall EWF due to changes in EWFs of individual phases may reflect corresponding variations in the overall mechanical properties of the material system.

Figure 5(b) illustrates another system in which finer domains of phases 1and 2 are densely distributed. In this case, the overall EWF is dominated by the phase which has a higher EWF, depending on size and spacing between adjacent domains of the higher EWF phase. This has been demonstrated by a recent study on TiC-Ni composite coatings with two sizes of TiC particles on micron and nanometer scales^27^. Effects of second phase’s size and morphology on the interfacial coherency and thus properties of a two-phase material are also reflected by EWF^27^,^28^. As observed in the study on TiC-Ni composite coatings, lower interfacial coherency (caused by large TiC particles embedded in Ni matrix) resulted in lowered EWF or electron localization at the interface. Such lowered EWF at interfaces deteriorates the resistance of materials to mechanical and electrochemical attacks^29^,^30^. However, the situation is reversed when the particle size is on nano-scale with elevated interfacial coherency^27^,^28^.

Based on collected information from current and previous studies, it appears that the overall EWF integrates work functions of individual microstructure constituents (phases, interfaces) and their mutual influence or synergy. Such an integrated EWF may reflect material properties to a certain degree. However, more studies are needed for comprehensive understanding of the microstructure-EWF-property relationships. Relevant research has been underway.

### Effects of the second phase, FeNi_3_, on the properties of Ni-added X70

**Figure 6:**
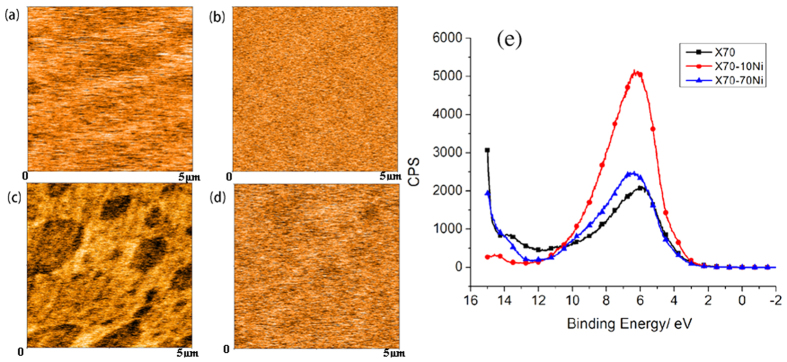
EWF maps of (**a**) X70, (**b**) X70-10Ni, (**c**) X70-30Ni, (**d**) X70-70Ni, respectively, obtained by nano-Kelvin probing. Darker regions have lower work functions and brighter regions show higher work functions. The work function distribution of X70, X70-10Ni and X70-70Ni are approximately homogenous. X70-30Ni has two different groups of domains with different work functions. (**e**) CPS ~ binding energy curves of X70, X70-10Ni and X70-70Ni. The area under a curve is proportional to the number of free electrons in the sample. The number of free electrons is highest for X70-10Ni, and those of X70 and X70-70Ni are close.

**Figure 7:**
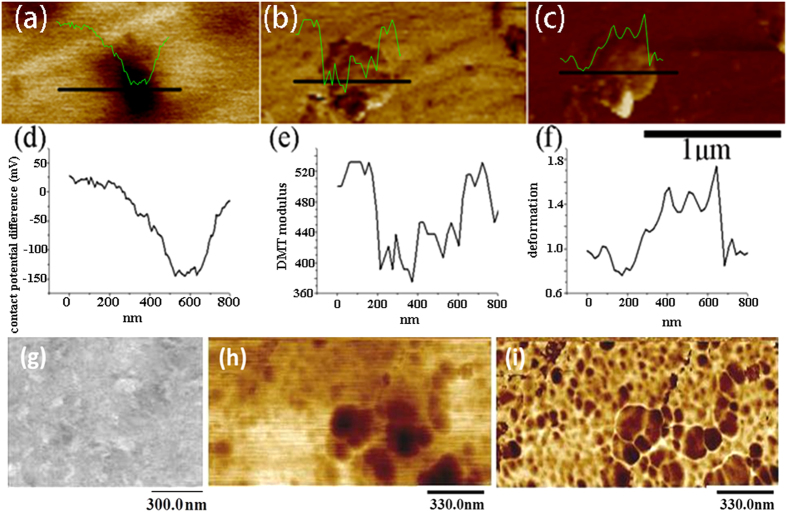
(**a–c**) show *in situ* EWF, elastic modulus and deformation maps of X70-30Ni with inserted line property profiles, obtained using a multi-mode AFM. In the EWF map (**a**) the darker domain (FeNi_3_) has a lower work function and brighter regions show higher work functions. As demonstrated by the elastic modulus (**b**) and deformation (**c**) maps, the darker domain is softer than the brighter regions. (**g**) A SEM image of X70-70Ni, (**h**) a EWF map and (**i**) corresponding elastic modulus map of the X70-70Ni sample.

As shown earlier, the formed FeNi_3_ phase resulted in a deviation from the trend of changes in EWF with %Ni, observed for isomorphous solid solutions in which solute atoms are homogeneously dispersed in the host lattice. More detailed analysis was made to investigate influences of this second phase on the Ni-added X70 steel. A multimode atomic force microscope (AFM) with a nano-Kelvin probe was used to map variations in local EWF of samples with different concentrations of Ni. Figure 6 illustrates work function distributions of X70, X70-10Ni, X70-30Ni and X70-70Ni. As shown, the first two alloys show approximately homogenous EWF distributions, which is consistent with the XRD patterns in which the two alloys only have a single phase. However, in the X70-30Ni sample there are distinguishable domains with lower work function. The work function of darker domains is approximately 0.16 eV lower than that of brighter domains. Since the EWF of X70-70Ni is about 0.2 eV lower than that of X70-10Ni and the former is basically FeNi_3_, it is believed that the darker domains in Fig. 6(c) are FeNi_3_. While the brighter area is the *α*-Fe matrix with saturated dissolved Ni addition, whose composition should be close to X70-10Ni. The X70-70Ni sample appeared to be homogenous. However, a closer view (see Fig. 7) shows that there are many nanometer-scaled FeNi_3_ domains in the sample. Since the composition of this sample is close to that of FeNi_3_, the nucleation rate of FeNi_3_ could be sufficiently higher, leading to the formation of many FeNi_3_ nano-domains in the sample.

We also examined the samples under a scanning electron microscope. However, due to limited resolution, the nano-scaled FeNi_3_ domains are too small to be clearly viewed under SEM. A SEM image of X70-70Ni is still included in Fig. 7, from which nano-scaled domains are more or less visible.

To further analyze variations in EWF, we examined the samples using Ultraviolet Photoelectron Spectroscopy (UPS), from which their electron band structure and valence electron density can be determined^31^. Ultraviolet light shines at the sample using a Helium lamp emitting at 21.2 eV (He radiation). The photons of low energy cannot excite and emit core electrons out of sample but have access to the valence band or can emit valence electrons. Thus, UPS can be used to analyze the valence band structure of materials and the low binding energy part of the spectrum mirrors the electron density of state in first-order approximation. The integral of the spectrum is the total number of electron states of the materials, and each state is occupied by two electrons. Since electron is a kind of fermion, the integral of the spectrum multiplied by Fermi-Dirac distribution function equals to the number of electron states if temperature is higher than 0K^1^. Fig. 6(e) illustrates the count per second (CPS) ~ binding energy curves of X70, X70-10Ni and X70-70Ni. The area under a curve is proportional to the number of free electrons in the sample. As shown, the number of free electrons increases when 10%Ni is added to X70, but it decreases when more Ni is added. The number of free electrons of X70-70Ni is similar to that of X70. The decrease in EWF of X70-10Ni when more Ni added is attributed to the formation of the intermetallic phase, FeNi_3_. The change in electron density measured by UPS may explain the trend of variations in EWF, since EWF is proportional to the free electron density, where the number of free electrons per atom is equal to the nominal valence of the element or close to its number as expressed in eq.(1)^32^,^33^,


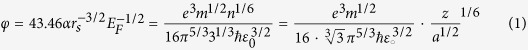


where r_s_ is the electron density parameter, E_F_ is Fermi energy, *α* is a constant, n is the equilibrium valence electron density, z is the number of valence electrons, *a* is the lattice constant, m is the electron mass, and *ε*_0_ is the vacuum permittivity, ћ; is Planck constant, and e is the elementary charge.

When Ni is added to Fe without forming the second phase, it brings more free electrons to the system and consequently raises the free electron density, leading to a higher overall work function. As a result, the interaction between electrons and nuclei is enhanced, rendering the material stronger. When too much Ni is added with the formation of intermetallic phase, FeNi_3_, previous free electrons may not all serve as valence electrons. It is known that electrical conductivities of intermetallic compounds can be much lower than those of corresponding individual elements and some of them may behave like semiconductors^34^,^35^. The decrease in the free electron density could be ascribed to possible change from the metallic bond to one(s) with less metallic characteristics or a mixture of metallic and covalent/ionic bonds. In the present stage, it is not very clear why FeNi_3_ has a lower work function. However, no matter what is the bond type or whether atoms are bonded by sharing electrons or by electrostatic interactions, if electrons are easier to be taken out from a solid, the stability of its mechanical state is lower, corresponding to weaker atomic bonds that are easier to be broken.

Local electron work function, modulus and deformation of a X70-30Ni sample were mapped simultaneously using the multi-mode atomic force microscope in order to evaluate local properties of the sample consisting of FeNi_3_ phase domains and the matrix. As illustrated in Fig. 7(a–c), X70-30Ni shows two different regions with different work functions. The dark domain having a lower EWF in Fig. 7(a) is softer than the matrix, evidenced by its lower elastic moduli and larger deformation (Fig. 7(b,c)).

In order to support or further confirm the experimental observations, mechanical properties of *α*-Fe, Ni and FeNi_3_ were calculated using the first-principles method.

*α*-Fe has a BCC structure and the other two have FCC structures. FeNi_3_ is an ordered phase with Fe atoms occupying the corner positions and Ni atoms located in face centers^23^. The calculated elastic constants are consistent with the experimentally measured values^23^, both of which are given Table 2. In Table2, bulk modulus B of polycrystalline samples was also calculated from the elastic constants.


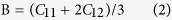


Unlike the bulk modulus, Young’s modulus and shear modulus are more crystallographic orientation dependent. Thus, they are not calculated here for comparison purpose in order to prevent misleading information. Experimentally measured bulk modulus is given in Table 2 as well, which is in good agreement with the calculated one. As shown, Ni is more stiff than both *α*-Fe and FeNi_3_. *α*-Fe has medium bulk modulus, higher than that of FeNi_3_. This is consistent with the order of their work functions. When Ni with a higher work function is added to X70 steel, the overall work function increases. However, the situation is reversed when FeNi_3_ forms, which exhibits a lower EWF. The correlation between EWF and mechanical properties, including Young’s modulus, hardness and calculated bulk modulus and elastic constants, has been well demonstrated by this Fe-Ni alloy system.

**Figure 8:**
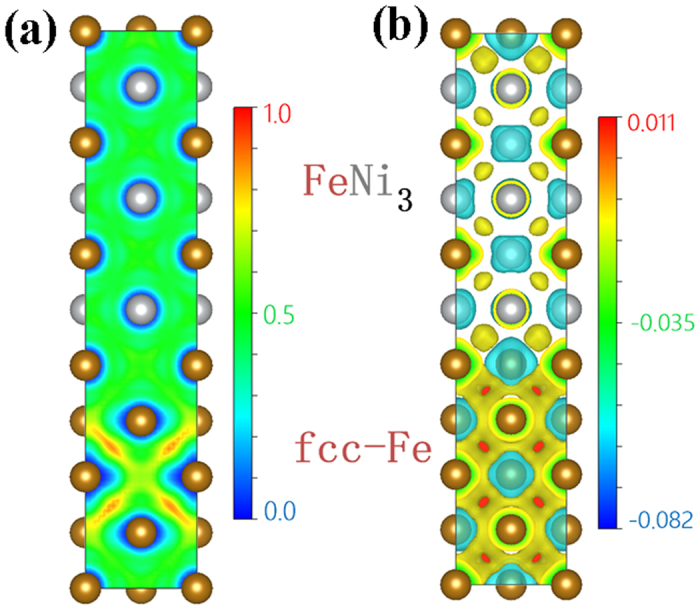
(**a**) Electron localization function (ELF) and (**b**) electron-density difference of Fe & FeNi_3_ supercell.

In order to understand why FeNi_3_ has a lower work function corresponding to lowered free electron density, we calculated variations in electron localization function and electron density across a *α*-Fe/FeNi_3_ interface using first-principles method. Such variations help view the difference in the degree of electron localization between *α*-Fe and FeNi_3_ phase and further understand the strengths of Fe-Fe bond and Fe-Ni bond in the FeNi_3_ phase. However, due to different crystal structures and large difference in lattice constant between BCC-Fe and FCC-FeNi_3_ as well as lack of relevant information on Fe/FeNi_3_ interfaces, it is difficult to construct an interface between these two phases, which can be used to perform convergent calculation. Thus, we study the interface between FCC-Fe and FCC-FeNi_3_ (see Fig. 8) with attention to variations in electron localization and density. Although the obtained information may not precisely reflect the interfacial situation, one may still see how the degree of electron localization changes when Fe and FeNi_3_ are adjacent to each other. From the calculation, one may see that electrons are more localized in Fe than FeNi_3_ and the former has its electron density larger than that of FeNi_3_. The calculations demonstrate that Fe has its ELF and electron density higher than those of FeNi_3_ phase. The calculation indicates that the Fe-Fe bond is stronger than that of Fe-Ni bonds in the intermetallic phase, FeNi_3_.

Electron localization function (ELF) is a simple measure of electron localization in atomic and molecular systems. It is a property of the same-spin electron pair density which represents the likelihood of finding an electron in the neighborhood space of a reference electron located at a given point and with the same spin. ELF 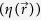 is expressed as^36^,^37^


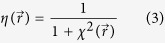



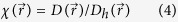


where the ratio 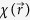 is a dimensionless localization index that expresses the electron localization with respect to the uniform electron gas as reference. The smaller the probability of finding a second like-spin electron near the reference point, the more highly localized is the reference electron and higher ELF. As shown in Fig. 8(a), ELF in Fe region is higher than that in FeNi_3_ region, indicating that the electrons in Fe are more localized or more tightly confined, corresponding to stronger Fe-Fe bond in contrast with those in FeNi_3_ phase. This explains why pure Fe has higher Young’s modulus and EWF than FeNi_3_ and why the modulus and EWF decrease when the intermetallic phase FeNi_3_ is formed as more than 10% Ni is added to X70.

We also calculated the electron-density difference, which is defined as the difference in the electron density between a Fe/FeNi_3_ supercell and isolated Fe or FeNi_3_ supercell. This parameter reflects the electron density of the system and related bond strength. As illustrated in Fig. 8(b), the electron density of Fe supercell is higher than that of FeNi_3_ supercell, which is consistent with the calculated electron localization function shown in Fig. 8(a) and experimentally measured electron density from UPS analysis, work function and elastic modulus illustrated in Figs 1 and 6(e), respectively.

## Conclusions

In conclusion, correlations between the electron work function and mechanical properties were investigated using Ni-added X70 steel as a sample material. It was demonstrated that adding Ni having a higher electron work function brought more “free” or valence electrons to the steel, increasing the overall work function of the material. Young’s modulus and hardness increased correspondingly. However, the free electron density and work function decreased as %Ni was continuously increased with the formation of an intermetallic phase, FeNi_3_. This softer phase resulted in lowered work function and corresponding deterioration in the overall mechanical strength.

The changes in Young’s modulus and hardness show trends similar to that of change in EWF with respect to %Ni no matter whether or not the second phase (FeNi_3_) is present. The similar trends suggest that the overall mechanical behavior is correlated with the overall EWF to a certain degree, although the former is microstructure dependent.

This study demonstrates that electron work function is a promising parameter, providing supplementary clues for element selection and material tailoring. It is expected that this simple but fundamental parameter may lead to the development of new methodologies or supplementary approaches for designing and tailoring metallic materials on a feasible electronic base.

## Methods

### Experimental details

**Figure 9:**
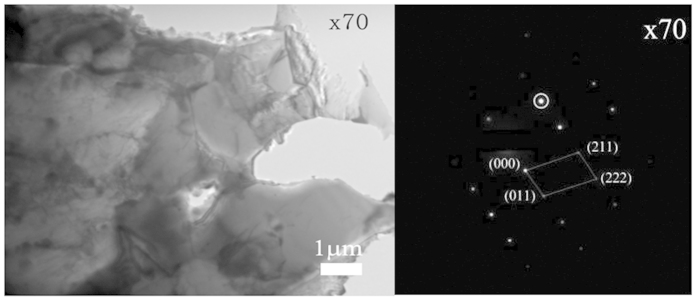
TEM image and diffraction pattern of the base alloy, X70 steel, which contains mainly ferrite with little cementite.

X70 steel (a common pipeline steel) contains only 0.066% C and very minor elements (Mn, Cr, Si, P, S) within their solubility. This steel, in a form of nearly iron with little cementite (see a TEM image of X70 in Fig. 9), is used as a base material for this study. X70 samples containing different amounts of Ni addition were made using an arc melting furnace, annealed (homogenization treatment) in a tube furnace at 600 °C in an Ar atmosphere for 3 hours followed by furnace cooling. The samples were polished using 400, 600, 800, 1200 grit sand papers successively and finally polished in a solution containing diamond particles of 1 μm in diameter. The polished surface was cleaned using an ultrasonic cleaner with reagent alcohol for 3 min. Right after drying, the work function of the surface was measured using a Scanning Kelvin Probe (KP Technology Ltd. UK) in order to minimize the influence of contamination and oxidation on EWF measurement. During the measurement, a surface under study was scanned by the Scanning Kelvin Probe over an area of 16 *μm* × 16 *μm* which covered 25 points for EWF measurement. At least three different regions on each sample and two samples for each alloy were analyzed for statistically reliable results. Young’s modulus and hardness of the samples were measured using a micro-indenter (Fisherscope H100C Micro hardness Measurement System, Fisher Technology, Germany). A cone-shaped diamond indenter tip was used and the load was applied at a rate of 30 mN/s up to 600 mN. Each sample was measured at least five times. Macro-hardness of the samples was measured using a Mitutoyo hardness tester under a load of 15 kg with a pyramidal indenter. Each hardness value is an average of 5 measurements made at randomly selected locations on each sample.

Crystal structures of the samples were analyzed with X-Ray Diffraction (Rigaku XRD Ultimate IV; Cu *kα* radiation). Surface morphological observation and local work function analysis were carried out using a multi-mode atomic force microscope with the capability for *in situ* measuring or mapping EWF, elastic modulus and deformation simultaneously. The electron density of the samples was analyzed by Ultraviolet Photoelectron Spectroscopy (UPS, Axis Ultra Spectrometer, Kratos Analytical). The UPS measurement was carried out in vacuum in order to prevent the influences of adsorption and contamination on the measurement. Before UPS tests were performed, sample surfaces were sputtered by argon ions in order to eliminate possible surface contamination. A multimode atomic force microscope (Bruker, US) was used to analyze local EWF, magnetic properties of phases and modulus of different phases/domains.

### Simulation details

All energy calculations were carried out using the density functional theory (DFT) implemented in the Vienna Ab initio Simulation Package (VASP)^38^,^39^,^40^ with the projector-augmented wave (PAW) potential^41^. The generalized gradient approximation (GGA) with the exchange-correlation functional of Perdew, Burke and Ernzerhof (PBE)^42^ was employed. K-points sampling using Monkhorst-Pack^43^ with *k*-mesh of 21 × 21 × 21 was sufficient for structure optimization. A finer *k*-mesh (25 × 25 × 25) appeared to be optimal for calculating the elastic constants. The *K-mesh* of 25 × 25 × 1 was applied in supercells calculations. The elastic constants were calculated from the energy variation introduced by applying very small strains to the lattice in equilibrium. For a given strain, all internal atomic coordinates were fully relaxed while keeping the deformed lattice fixed. The elastic energy, 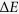, of a crystal can be expressed as^44^:


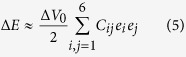


where Δ*V*_0_ is the volume of a undistorted crystal unit cell, the vector *e* = (*e*_1_, *e*_2_, *e*_3_, *e*_4_, *e*_5_, *e*_6_) represents the applied strain, and coefficients *C*_*ij*_ are elements in the elastic-constant matrix. Δ*E* and Δ*V*_0_ were fitted with a quadratic polynomial to obtain the relevant elastic constants. Since the systems under study are cubic structures, there are only three independent elastic constants, *C*_11_, *C*_12_ and *C*_44_.

## Additional Information

**How to cite this article**: Lu, H. *et al*. Electron work function–a promising guiding parameter for material design. *Sci. Rep*. **6**, 24366; doi: 10.1038/srep24366 (2016).

## Figures and Tables

**Table 1:** Hardness of X70 samples with different concentrations of Ni.

Sample	Hardness (HV)
X70	60.8 + /−1.1
X70-2Ni	65.8 + /−0.8
X70-5Ni	74.3 + /−0.3
X70-10Ni	74.5 + /−1.1
X70-15Ni	72.9 + /−3.3
X70-20Ni	72.6 + /−1.2
X70-30Ni	40.0 + /−0.2
X70-50Ni	44.2 + /−0.8
X70-70Ni	38.4 + /−3.0

Hardness was measured using a macro-indenter under a load of 15kg.

**Table 2:** Elastic constants and elastic moduli of Fe, Ni and FeNi_3_ from first principles simulation and experiments; the experimental data are cited from a reference^23^.

	Fe(BCC)		Ni(FCC)		FeNi_3_(FCC)	
	Exp.	Cal.	Exp.	Cal.	Exp.	Cal.
C_11_	242.0	268.1	246.5	268.7	230.4	237.2
C_12_	146.5	144.2	147.3	150.9	144.4	136.9
C_44_	112.0	83.4	124.7	129.9	119.2	114.7
B (GPa)	178.3	185.5	180.4	190.2	173.0	174.0
